# Outbreak of New Delhi Metallo-β-lactamase-producing Escherichia coli in a Neonatal Intensive Care Unit, New York State

**DOI:** 10.1017/ash.2024.284

**Published:** 2024-09-16

**Authors:** Jane Greenko, Sarah Kogut, Kailee Cummings, Shannon Morris, Elizabeth Nazarian, Catharine Prussing, Karen Southwick, Emily Lucrezia, Warangkana Sangchan

**Affiliations:** New York State Department of Health; Wadsworth Center, NYSDOH; CDC Foundation; New York State

## Abstract

**Background:** Outbreaks of carbapenemase-producing (CP) organisms (CPOs), including carbapenem-resistant Enterobacterales (CRE), in neonatal intensive care units (NICUs) are not well documented. The Centers for Disease Control and Prevention (CDC) identifies CP-CRE as an urgent threat to United States (US) healthcare facilities. Wadsworth Center, the New York State (NYS) Department of Health’s (NYSDOH’s) public health laboratory, participates in CDC’s Antimicrobial Resistance Laboratory Network to provide CPO identification, characterization, and surveillance. NYSDOH investigated an outbreak of CP-CRE Escherichia coli (E. coli) infections in NICU patients reported by one hospital. **Method:** Hospital A reported a CRE E. coli outbreak in their NICU to NYSDOH, as required by NYS Sanitary Code. In response, epidemiologists reviewed case data, conducted case finding, and provided infection control guidance to the hospital. Hospital A continued NICU clinical surveillance and conducted colonization screening to detect additional cases of CRE E. coli. The Wadsworth Center Antimicrobial Resistance Laboratory Network tested isolates from affected patients for CP genes and performed whole genome sequencing (WGS) to determine the CP gene variant, multilocus sequence type (MLST), and relatedness by mutation event (ME) analysis. NYSDOH epidemiologists assessed Hospital A’s infection control practices in affected areas and provided recommendations. **Result:** Hospital A identified two CRE E. coli infections in NICU patients with overlapping admissions in June-July 2023. Retrospective surveillance identified a third CRE E. coli case in an adult medical intensive care unit patient on admission to Hospital A in June 2023, with prior hospitalization April-May 2023. WGS analysis identified the blaNDM-5 gene in all three CRE E. coli patient isolates. The two NICU patients’ isolates had the same MLST (361/650) and differed by 9 MEs, indicating relatedness to each other and not the adult patient’s (MLST 167/2). NICU patient colonization screening identified no additional blaNDM-5 E. coli cases. NYSDOH’s NICU infection control assessment found that both cases were in adjacent isolettes within three feet of each other. Clean isolettes, equipment, and supplies for new admissions were stored in the clinical care space, not in a separate clean area. **Conclusion:** CP-CRE is an urgent threat to US healthcare facilities, including hospital NICUs. Though the incidence and prevalence of CP-CRE blaNDM-5 E. coli are not well-defined in NY, single healthcare-associated cases in NICU populations represent an outbreak. The Wadsworth Center Antimicrobial Resistance Laboratory Network’s contributions complement traditional epidemiologic surveillance and investigation methods to provide more specific, comprehensive infection

